# Exploring culinary medicine as a promising method of nutritional education in medical school: a scoping review

**DOI:** 10.1186/s12909-022-03449-w

**Published:** 2022-06-07

**Authors:** Jacqueline Tan, Levi Atamanchuk, Tanish Rao, Kenichi Sato, Jennifer Crowley, Lauren Ball

**Affiliations:** 1 Griffith University, Gold Coast, Australia; 2 University of Auckland, Auckland, New Zealand

**Keywords:** Culinary medicine, Nutrition, Nutrition care, Nutrition education, Culinary education, Lifestyle education, Lifestyle medicine, Food as medicine

## Abstract

**Background:**

Dietary modifications are considered a first-line intervention for chronic disease management, yet graduating doctors still report not feeling competent to counsel patients on their diet. Research has focused on methods to address this shortfall in physician competency, including culinary medicine. Culinary medicine is an approach to education that involves hands-on food and cooking learning experiences to equip participants with tools for improving the nutrition behaviour and health of their future patients. Despite positive findings in the efficacy of these interventions, they differ markedly in approach and target, which therefore fails to provide adequate evidence that could serve to guide future culinary medicine interventions.

**Objective:**

A scoping review to synthesize the existing literature on culinary medicine interventions that are offered during medical training.

**Methods:**

Online databases were used to identify literature published prior to April 2022 that involve a hands-on culinary medicine component to nutrition and examine academic impact, feasibility and acceptability.

**Results:**

*Twenty-four studies met the eligibility criteria.* Despite promising gains in nutrition knowledge, confidence and high acceptability of the programs, large variations exist in delivery method, setting, and course content between programs. There is a lack of program cost reporting and long-term follow up of participants, inconsistent evidence for improved nutrition attitudes amongst participants, as well as geographically limited adoption of such programs.

**Conclusions:**

The findings of this research demonstrate a clear increase in interest in the use of hands-on culinary medicine programs as educational tools, evidence of feasibility in implementation, and improved student nutritional knowledge, skill and counseling compared to a traditional didactic curriculum. The quality of culinary medicine research studies is increasing and the aims of research are narrowing to focus on how culinary medicine can positively impact medical education. The findings from this review will aid in legitimising culinary medicine as an effective delivery method of nutritional education in medical programs.

**Supplementary Information:**

The online version contains supplementary material available at 10.1186/s12909-022-03449-w.

## Background

Chronic disease accounts for 41 million deaths annually, equivalent to 71% of all deaths globally. Poor dietary habits and lifestyle behaviours are leading modifiable risk factors for chronic diseases [1]. Nutrition care is the practice of improving the nutrition behaviour and health of patients conducted by health professionals [2]. As a result, nutrition care that supports patients to eat well is considered as an essential, first line step in preventing and managing chronic disease [1]. However, doctors report not feeling equipped to provide adequate nutrition care to patients, despite their acknowledgement that nutrition is a useful and necessary part of patient care [2, 3].

Culinary medicine is an emerging field of education related to meal planning, preparation and cooking skills in combination with counseling practices to promote health behaviour change in patients/clients [4]. Interest in culinary medicine has rapidly increased among many medical schools across the United States and other countries as a potentially low-cost, high impact strategy to equip students with practical nutrition skills that can be employed for nutrition care in clinical settings [5]. Several pilot culinary medicine programs have occurred [5] which theorise that culinary medicine initiatives completed in undergraduate medical programs may produce doctors that feel more confident providing nutrition care to their patients [6, 7]. These programs have demonstrated significant and positive impact on medical student’s attitudes, knowledge, and competencies with practical, hands-on culinary skills and nutrition knowledge, which ultimately supports their patient counseling [8]. Many aspects of culinary medicine pilot programs are unique and wide differences exists in structure, duration, and setting. While these programs provide a wide range of suitable information, they are not helpful for recommending a standardized implementation of culinary medicine in medical programs. A recent scoping review by Asher et al., 2021 explored literature that provides culinary and nutrition training to and by health, education, and culinary professionals. This study demonstrated that the feasibility and outcomes of culinary medicine programs warrant further investigation as there is still insufficient evidence of the impact these programs on practitioners to promote health behaviour changes in patients [4] and the viability of culinary medicine [9]. This scoping review aims to synthesise the existing literature on culinary medicine offered to medical students within their four years of medical school education.

## Methods

### Overview

This review critically synthesised literature examining culinary medicine programs offered to medical students in the setting of a four year medical program. The review was informed by PRIMSA methodological guidelines. The protocol was registered with PROSPERO (ID: CRD42020210766) and the reporting followed PRISMA guidelines [10].

This scoping review was initially registered with PROSPERO as a systematic review. This review was changed into a scoping review because it offers a wide exploration of culinary medicine research and its characteristics and gaps, rather than answer the specific question of whether culinary medicine programs cause, and adequately assess, change in medical students’ attitudes, skills and knowledge of nutrition.

### Search strategy

An electronic literature search was conducted initially in June 2020 with the support of an academic librarian. A second electronic literature search, identical to the initial search, was conducted in April 2022 to provide a more comprehensive review, before project completion. Computer-based searches were conducted within MEDLINE (EBSCO*host)*, SCOPUS, Web of Science, ProQuest (central), Taylor and Francis online, and SAGE journals. The search string entered for MEDLINE was (“Culinary Medicine” OR “Nutritional Medicine” OR “Culinary Education” OR “Food as Medicine”) AND (“medical”) AND (“Student” OR “Program” OR “Education” OR “Training”) with the “scholarly (peer reviewed) journals filter selected. The search strategy was adapted to each database to accommodate for variation in database search engine function. Filters to exclude articles that were not peer-reviewed were used if the database provided that feature. The search strings and filter settings for each database used have been provided in Additional file 1.

### Selection criteria

All full-text, peer-reviewed journal articles published prior to 22 April 2022 were eligible for screening. This eligibility criteria was enlisted to ensure that this scoping review synthesizes the existing literature on culinary medicine that explores the feasibility of culinary medicine programs, which may offer evidence-based advice for future interventions.

- *Population:* Medical students, or medical graduates if they received the intervention during medical school.
- *Intervention*: Any form of culinary medicine education (curriculum or elective) received during medical school. Culinary medicine was considered to be used if the article referred to the activity in that way, or if a practical/hands-on cooking related teaching experience occurred.
- *Comparison:* Studies must have had a control group (difference between groups) or baseline measures (report a difference over time).
- *Outcome Measures:* Changes in medical students' nutritional knowledge, skills, or attitudes were considered relevant.

Preliminary title and abstract screening occurred on 50 records by importing the details into Microsoft Excel and screening by LB, TR, KS, LA, and JT to establish interrater reliability. Interrater reliability using the selection criteria was 98% and deemed sufficiently high to progress to screening.

**Fig. 1:**
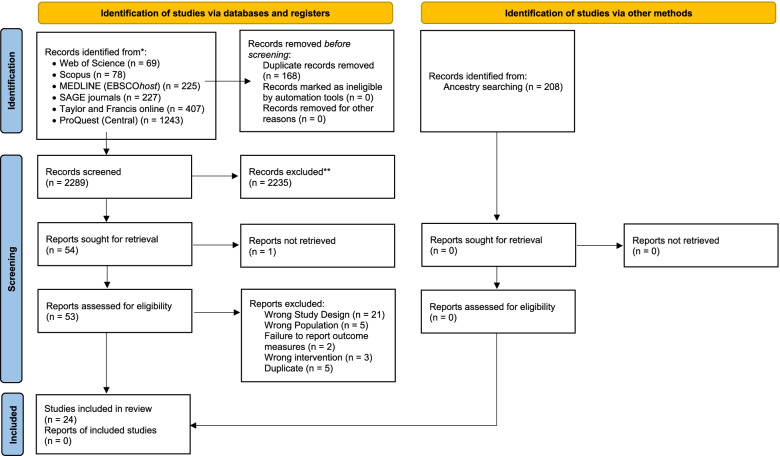
PRISMA 2020 flow diagram

All records were then imported to Covidence for screening and duplicates were removed upon import. The titles and abstracts for all articles were independently screened twice, by any two of the researchers (TR, KS, LA, LB and JT), facilitated by the Covidence software system. Full-text articles were retrieved and screening to be included in our final review was performed by one researcher and verified by a second researcher (TR, KS, LA or JT). Conflicting screening results were resolved during regular team meetings. Additional articles were identified via ancestry searching and were manually added for screening using the same procedure facilitated by Covidence. The screening process is outlined in Fig. 1 using the PRISMA 2020 diagram [10].

### Data charting

Data charting was performed independently by one researcher (TR, KS, LA, or JT) and confirmed by another researcher (TR, KS, LA, JC, or JT). Variables for which data was sought included study characteristics such as setting, population, aim of research, and description of program, “course description”, intervention, outcomes, and notable findings (Table 1 and 2). Trends in data were also sought in the format of a yes/no table. Quantitative and qualitative studies were included in this scoping review and were analysed using meta-synthesis, an interpretation of results to offer novel information regarding the viability of culinary medicine in medical education.

**Table 1:** Study Descriptions

Authors	Study Design	Setting	Population	Aim(s) of research	Course Description
Monlezun et al. (2015)	Cross-Sectional	University Teaching Kitchen and various Community Centres	Medical students; *n* = 627; Attrition NR; Any year	To investigate superiority of "stimulation based medical education with deliberate practice" style hands-on cooking and nutrition education over traditional clinical education for preventive medicine in a large sample of medical students	8 session elective (28 h total): consisting of PBLs and 0·5 h pre-module video lecture, pre-module quizzes and quiz review; 1·5 h hands-on cooking. Program continues into interdisciplinary seminars for Third year medical students and a four week away rotation for Fourth year students
Dreibelbis & George (2017)	Qualitative	Senior Centre Teaching Kitchen	Medical students; *n* = 4; Attrition = 0%; 4th year	To partner Penn State College of Medicine with a senior center and launch a pilot culinary medicine elective with a focus on involving older community residents as intergenerational mentors	8 session elective: (3 h each) in a 2 week program consisting of in-person case-based discussions for 1 h; collaborative cooking for 2 h with elder mentor, pre-class readings, pre-session standardised quizzes and 2 hands-on culinary skills lessons with professional chef facilitator
Jaroudi et al. (2018)	Mixed Methods	University Lecture Hall and University Cooking Lab	Medical students; *n* = 20; Attrition = 15%; 1st and 2nd year	To develop and implement a culinary medicine elective into Texas Tech Universities Medical Program to equip students with confidence in knowledge and skills in culinary medicine concepts and to improve physician's integration of preventative medicine into patient care proficiency	Elective course: duration or schedule NR; consisting of four didactic sessions and four interactive kitchen-based labs during the semester; delivered by local chefs, other medical students, and hospital dietitians
Monlezun et al. (2018)	Cross-Sectional	Varied Based on Institution	Medical students; *n* = 3248; Attrition NR	To determine if hands-on cooking and nutrition education for medical students can have inferred causality for improved student competencies and attitudes towards providing patients with nutrition education, students' own diet and if such a program could be scalable	8 session elective (22 h total): consisting of 0·5 h pre-class online lecture videos, a 1·5 h hands-on cooking, and a 0·75 h post-class PBL session; delivered through CHOP instructors (variable based on institution implanting the program
Hauser et al. (2019)	Qualitative	Offsite Commercial Kitchen	Medical students, Physician assistant students; *n* = 36; Attrition NR; Any year	To produce a Teaching Kitchen Elective for medical students that focuses on hands-on culinary techniques, dietary counseling for patients to overcome medical graduates' lack of confidence in nutrition counseling, self-care and culinary skills	8 session elective (2 h each): consisting of weekly pre-class videos and handouts; hands-on culinary skills lessons, case-based round table group discussions and recipe-based assignments; delivered by 2 physician-chefs, an executive chef, external professors specialising in medical education, nutrition and prevention research and a variety of specialty faculty members faculty members
Lawrence et al. (2019)	Case Study	University Food Lab	Medical students, nutrition students, and medical residents; *n* = 48; Attrition = 27%	To use a culinary medicine course to strengthen teamwork between nutrition and medical students using a collaborative environment to develop better understanding of their respective scopes of practice and establishing groundwork for effective interprofessional communication	25 h nutrition elective: consisting of 5 sessions per semester, using 5 modules from Goldring Center for Culinary Medicine at Tulane University: includes online didactic components, and 3 h in person interactive team component; taught by interdisciplinary faculty members, chef/dietitian, clinical dietitian, and family medicine physician
Pang et al. (2019)	Mixed Methods	Non-Profit Teaching Kitchens	Medical students; *n* = 15 (2016), *n* = 16 (2017), *n* = 16 (2018); Attrition for all years = 0%; 2nd year	1) To develop a hands-on approach to medical nutrition education centred around teamwork. 2) To create innovative ways with food and cooking for students to connect with and understand health disparities faced in a county community population. 3) To increase medical students’ confidence counseling patients with chronic disease to make dietary change	6 session elective (15 h total): consisting of 0·5 h of physiology and nutrition lessons, 1·5 h of hands-on cooking class, and 0·5 h of communal eating and session debrief; delivered by collaboration between a physician, a registered dietitian, and a chef
Ring et al. (2019)	Mixed Methods	Non-Profit Teaching Kitchen and Chicago Public Schools	Medical students; *n* = 9 (cohort 1), *n* = 12 (cohort 2); Attrition = 11% (cohort 1), Attrition = 0% (cohort 2); 1st and 2nd year	1) To evaluate the feasibility and acceptability of the culinary medicine elective by examining class attendance rates, retention, and qualitative and quantitative feedback about the course. 2) To examine preliminary efficacy of the elective in preparing medical students to counsel patients in successful behaviour change in nutrition and cooking. 3) To improve medical students’ own cooking and nutrition confidence, attitudes, and behaviours	Cohort 1: 6 elective in-class sessions facilitated by faculty and chef (~ 2·5 h each): consisting of didactic teaching, counseling and motivational interviewing practice, culinary instruction and group dinner (hours NR); PLUS post-hoc 4 session service component (duration NR) teaching elementary public-school children.; Cohort 2: 6 session elective facilitated by faculty and chef: consisting of pre-class videos (3–8 min), lecture and assignment (totalling 1–1·5 h) in-class simulated patient coaching, student-led research discussions, culinary sessions (hours NR); PLUS concurrent service component 4 sessions (duration NR) teaching elementary public-school children
Hennrikus et al. (2020)	Qualitative	University Lecture Hall	Medical students; *n* = 380; Attrition NR; 1st year	To vertically integrate basic science metabolic and immunologic pathways with clinical disease using nutrition in a constructivist educational model	13 week non-elective basic science course consisting of lectures, PBL, simulation sessions, review sessions and patient encounters culminating in competitive "cook off" where PBL groups prepared dishes for 1 of 5 specific disorders based on previous PBL cases
Patnaik et al. (2020)	Cross-Sectional	University Teaching Kitchen	Medical students; *n* = 4125; Attrition NR	To determine differences in efficacy between University of Texas Health Sciences Center implementation of practical culinary and nutrition curriculum compared to other participating CHOP schools	8 session elective (28 h total): consisting of 0·5 h pre-class lecture video, 0·5 h of case-based learning, 1·5 h of hands-on cooking, 0·75 h post class PBL session
Razavi et al. (2020)	Cross-Sectional	Varied Based on Institution	Medical students; *n* = 4215; Attrition NR	To assess the association between participation in kitchen-based nutrition education and Mediterranean dietary intake among medical students	8 session elective (32 h total): consisting of 4 h modules divided into 1 h online didactic program, 1·5 h in-kitchen team-based case studies and nutrition discussion, 1·5 h of hands-on cooking facilitated by physicians, chefs, and dietitians
Rothman et al. (2020)	Mixed methods	Clinic-Based Teaching Kitchen and West Philadelphia High Schools	Medical students; *n* = 31; Attrition *n* = 3%; 4th year	To report initial outcomes of a pilot nutrition and culinary medicine course targeting post-clerkship	8 session elective (2 h each) taught by a chef instructor, dietitians, physicians, and patients: consisting of briefing on relevant nutrition science (5 min), culinary instruction and demonstration by a chef (75 min), case discussion during eating of a meal (30 min), a concluding debrief of the session (10 min) and a capstone project involving food planning for mock patient and presentation. Additional service component included 8, weekly sessions teaching disease prevention practical nutrition to high school students (hours NR)
Lieffers et al. (2021)	Qualitative	University of Saskatchewan undergraduate foods laboratory	5 workshops run with different students from different health programs (including medical and nutrition students) *n* = 58, attrition NR, Participant’s year of program NR	To describe the implementation and evaluation of interprofessional culinary education workshops	A single, 3 h workshop; elective: Workshops focused on food security and Indigenous foods relevant for Saskatchewan. Each workshop began with a didactic session, followed by cooperative cooking with a professional chef in small groups in the university teaching kitchen
Magallanes et al. (2021)	Case Study	University of Texas Southwestern Medical Center	Two separate cohorts of 32, medical students; *n* = 64; attrition 6·25%; 1st year	1) How a culinary medicine elective course affected student counseling confidence, familiarity with evidence-based nutrition interventions, and understanding of the role of interprofessional engagement to address lifestyle-related disease and 2) To propose directions for future research regarding culinary medicine as a nutrition education strategy	3 h monthly meetings based on 8 modules from *Health meets Food* curriculum. Sessions included: small group discussions; practical food preparation skills for recipes relevant to the module; eating prepared food and discussion about the nutrition science, medical research, and patient care applications relevant to the module. Sessions co-facilitated by a medical doctor and a registered dietitian
Asano et al. (2021)	Case Study	West Virginia School of Osteopathic Medicine	Medical students; *n* = 42; Attrition 19%; all 4 years	To describe a culinary medicine elective course with a lifestyle modification focus and to evaluate the students’ perceived knowledge and attitudes in lifestyle medicine	2 week course. Students completed modules including video lectures, handouts and reading assignments and quizzes before completing faculty-facilitated application exercise sessions. Diabetes counseling lecture facilitated by DM education specialist. Nutrition ethics discussion, and clinical shadowing sessions included
Leggett et al. (2021)	Case Study	Touro University Nevada College of Osteopathic Medicine	Medical students; *n* = 16; attrition = 6.25%; all 4 years	To explore an alternative way to provide nutrition education without adding hours to the formal curriculum by (1) surveying student perceptions regarding current nutrition education, (2) surveying student interest in attending a nutrition elective, (3) selecting how the elective could best be delivered, and (4) running and assessing participants’ reactions to a short experimental version of the elective	3 session elective (2 hands-on culinary medicine sessions and 1 didactic session in between—total hours NR); The first culinary session dedicated to knife skills and culinary basics; the didactic session covered coronary artery disease and nutritional preventative measures followed by 2 clinical cases in the form of an essay assessment. Patient counseling sessions developed by a registered nutritionist: culinary sessions facilitated by culinary school teaching chef
Poulton & Antono (2021)	Mixed Methods	Online: University of North Carolina School of Medicine	medical students; *n* = 21; attrition = 14%; 3rd and 4th year	To explore the feasibility of running an online culinary medicine course	3, 75 min "live cooking workshops" and online course work elective; delivered remotely. Cooking done in participants own kitchens; class teachers’ credentials not specified
Hashimi et al. (2020)	Qualitative	A local market in a 'food desert'	Medical students; *n* = 117; 51% attrition; all 4 years of program (priority given to 1st year students	To determine the feasibility of applying the culinary medicine approach in an under resourced community setting; to evaluate student perceptions of the program value and to assess student self-rated learning of nutrition science, nutrition education, and social determinants of health	3 h training session: 2 h farmers market cooking demonstration; 1 h optional debriefing
Vanderpool et al. (2020)	Mixed Methods	Culinary Institute of the Carolinas Greenville Technical College	Medical students; *n* = 5; attrition = 0%; 1st and 2nd year	To evaluate the feasibility, efficacy, and efficiency of a culinary medicine course and to assess cooking knowledge, attitudes, behaviors, confidence, and self-efficacy pre- and post- course	2 weekly sessions of 10 modules over 5 weeks.. Each module consisted of: 3–4 h in class components taught by faculty; pre- and post- module homework and a chef led lab portion. held at the Culinary Institute of the Carolinas Greenville Technical College;
D'Adamo et al. (2021)	Mixed Methods	Teaching kitchen at the Institute for Integrative Health, a community-based non-profit, in Baltimore	Medical students at University of Maryland School of Medicine (UMSOM); *n* = 125; attrition = 4·8%; 1st year	To report on the implementation, curricular content, and mixed methods outcomes evaluation from the first cohort of first-year medical students at UMSOM, who received culinary medicine as a component of core medical student curriculum	2 session elective (6 h total) included: evidence-based nutrition instruction, group cooking of recipes based on the lecture concepts; eating the prepared meals together and discussing the potential application of the training to both patient care and the students’ self-care (1 h lecture provided by UMSOM faculty and 2 h cooking session covered basic training including kitchen safety; introduction to kitchen tools and knife skills and food preparation led by personnel from the Institute for Integrative Health, and Maryland University of Integrative Health
Kaye et al. (2018)	Qualitative	Wake Forest School of Medicine in Northern Carolina, and local YMCA community kitchen	Medical students at Wake Forest School of Medicine; *n* = 16; 1st year	To assess feasibility of scheduling and operating a student led lifestyle medicine curriculum and if students would participate in such a program (acceptability)	First 3 modules offered to entire cohort, integrated within the month-long orientation portion of the program. Modules 4–11 delivered to a cohort of 16 students (25·5 h total). Training included: hands-on components exploring grocery shopping on a budget; nutritional requirements to "build a healthy plate;” meal planning and patient motivation, Program was in partnership with the local YMCA and delivered by medical students with a "background of obesity treatment" and a faculty member
Flynn et al. (2019)	Case Study	Student lounge at Warren Alpert Medical School, Providence, RI	Medical students; 1st year *n* = 39 and 2nd year *n* = 5, at Warren Alpert Medical School; Attrition NR	To determine if a 4-week cooking program of plant-based, olive oil recipes would improve 1) diet and eating behavior in medical students and 2) practical nutrition knowledge	4, 30 min, elective sessions (2 h total); 15 min of recipe demonstration and preparation and 15 min of presentation of nutrition topic while food tasting; students encouraged to cook at home 3 dinners/week that followed similar recipes; facilitator roles NR
Kumra et al. (2021)	Case Study	Community church near primary care medical home, East Baltimore	Medical staff (medical assistants, office assistants, nurses, and physicians) and medical students; *n* = 34; attrition = 14·7%; (16 medical support staff and 12, 1st and 2nd year medical students, and 1 physician)	To determine if a culinary medicine curriculum delivered to a multidisciplinary team of primary care medical staff and medical students in a community setting would improve self-reported efficacy in nutritional counseling and if efficacy differed between participant roles	4 h interactive workshop delivered to medical staff and medical students within the neighborhood of a primary care medical home; Workshop included presentation on the principles of culinary medicine, motivational interviewing, nutrition education and counseling. Specifically, the 4 principles of motivational interviewing, engaging, focusing, evoking, and planning, were described, followed by a trip to the local food shops and then food preparation of 4 recipes while nutrition topics were reinforced; each workshop was led by a physician and registered dietician
Musick et al. (2020)	Case Study	Virginia Tech Carilion School of Medicine and culinary school teaching kitchen	Students from medicine, nursing, and physician assistant programs; *n* = 248 (medical = 84; nursing = 80; physician assistant = 84); attrition NR; year of program NR	1) To deliver Interprofessional education core competencies in an applied manner via culinary training and 2) to deliver basic concepts of nutrition to pre-clinical students and 3) to equip learners to apply culinary medicine skills in addressing the difficulties inherent in telling patients to “eat and cook healthy”	9, 3 h course sessions (27 h total). New culinary medicine track featured three components: 1) delivery of core content pertaining to clinical nutrition- included didactic presentations, small group learning sessions, simulation-based experiences, panel presentations, and a case-based learning activity whereby student teams worked through patient scenarios featuring nutrition as a prominent clinical issue; 2) team-based meal preparation and service—two meal preparation lab sessions including preparation of a collection of healthy recipes and 3) community outreach where participants taught nutrition concepts to children in various age groups at 7–8 different locations across the city. Sessions taught by an interdisciplinary team of a physician, nursing, physician assistant and health psychology faculty. 3 culinary school chefs guided students through the meal preparation and service processes

*n* Sample population, *NR* Not reported, *CHOP* Cooking Through Health Optimisation, *PBL* Peer-based learning

**Table 2:** Study Summaries

Authors	Outcome measures	Notable Findings
Monlezun et al. (2015)	**Quantitative**: Student diet and attitudes and competencies counseling patients on nutrition were compared using conditional multivariate logistic regression, as well as propensity score-weighted, and longitudinal panel analyses using 4 sets of the same 59 question survey	After fully adjusted conditional multivariate logistic regression, Tulane University’s CHOP significantly increased the pooled odds of total proficiency in overall competencies by 72% when compared to traditional clinical education (OR = 1·72, 95% CI: 1·54–1·92, *p* < 0·001)
Dreibelbis & George (2017)	**Qualitative**: Observations by researchers evaluating the interactions and experiences of students and elders, post-intervention written self-reflections by students and elders participating in the course	Participants shared the perception that elders had been able to successfully impart their lived experience and developed an understanding of the logistical and economic challenges patients face while making nutritional choices
Jaroudi et al. (2018)	**Quantitative**: Pre- and post-course Likert-scale surveys of culinary skill, knowledge of ingredients, knowledge of cooking techniques and ability to use kitchen supplies. Analysed using Mann–Whitney U test. A second pre- and post- course Likert scale survey and open-ended questions to assess nutrition knowledge and confidence, cooking habits, and factors that contribute to success of physician in promoting lifestyle changes. Analysed using Mann–Whitney U test **Qualitative:** A set of 3 open-ended questions were asked in the post-course survey about participant experience	A significant increase in students’ confidence in overall culinary skill level, knowledge of ingredients, knowledge of cooking techniques, and ability to use kitchen supplies (*P* = 0·002, 0·002, 0·0004, and 0·003, respectively). Nine participants reported improvement in their knife skills and eight students reported an improvement in their knowledge of dietary restrictions
Monlezun et al. (2018)	**Quantitative**: Propensity score-adjusted multilevel mixed effects multivariable regression showing medical students' improved competencies across 25 topics: All results are reported as fully adjusted Odds-Ratios. Analysed using two-tailed *P* value	Report of multi-site CHOP trial shows highest power study on culinary medicine program efficacy currently, with 4026 completed surveys across more than 45 medical schools indicating good generalisability. Significant improvements were seen for participants in all aspects of nutritional counseling competency and attitudes towards positive impact of nutritional counseling in clinical practice
Hauser et al. (2019)	**Qualitative**: Pre- and post-course surveys evaluating attitudes, knowledge and behaviours about healthy cooking, eating, motivational interviewing of patients focusing on making dietary behaviour changes, and general course feedback. Data and analysis not reported	Survey data and analysis were not presented in article. Authors suggest that preliminary findings indicate practical instruction of nutritional education is superior to traditional methods for teaching nutritional counseling
Lawrence et al. (2019)	**Quantitative**: Pre- and post-course surveys including team performance scale (TPS) (evaluating quality of team interactions) and 6-point Likert scale to rate frequency of participants engagement in each team-building process. Analysed using paired t test	Despite high pre-course TPS scores, post-course TPS scores for the entire group, medical students and residents, and nutrition students were significantly higher (*t* = -4·79, *P* < 0·001; *t* = -4·15, *P* = 0·001; and *t* = -2·71, *P* = 0·02, respectively)
Pang et al. (2019)	**Quantitative**: Pre- and post- survey assessing students' knowledge in nutrition, ability to identify various foods by visual inspection, and confidence in cooking and nutritional counseling **Qualitative**: Additionally, students provided course feedback on ways to improve the course and rated overall satisfaction with the class. Wilcoxon Signed Rank test used to analyse survey results	Among the three cohorts, all students self-perceived nutrition knowledge and confidence scores increased significantly. Students self-perceived food identification scores increased significantly in 2017 and 2018 only. Students rated overall course satisfaction at 4·87/5, 5/5 and 5/5 for 2016, 2017 and 2018 intakes, respectively
Ring et al. (2019)	**Quantitative**: Pre- and post-course survey on self-perceived competency in nutritional counseling using Conner and colleagues, Perceived Competency in Obesity Counseling Scale (PCOC), attitudes toward nutrition in patient care using Nutrition in Patient Care Survey (NIPS), cooking confidence using "The Cooking with the Chef Evaluation Instrument" (Cohort 1), and Cooking and Food Skills Measure (Cohort 1), and personal dietary habits using PrimeScreen Dietary Screening Tool. Post-course only, survey on likelihood of course recommendation. Analysed using ANOVA and Bonferroni corrections, calculations of mean, standard deviation, confidence interval and *P*-value of changes over time **Qualitative**: Open ended questions with prompts	Across two pilot cohorts, retention rates and attendance rates were more than 89% and 96% respectively, with mean recommendation scores of 6·25 and 6·67 out of 7, indicating good feasibility and acceptability. Notable methodologies include use of peer-reviewed competency scales for participants self-evaluations. Significant improvements in students’ counseling competencies in nutrition and obesity reported. No significant or mixed results reported for attitudes to nutrition counseling, culinary techniques and personal dietary habits
Hennrikus et al. (2020)	**Qualitative**: Post-course evaluation of strengths and weaknesses of individual classes and the course, using open-ended, free text, voluntary responses. Thematic analysis of qualitative data using grounded theory: data managed using ATLAS.ti 8.0 software	Qualitative results described three major themes: increased relevance of basic science to real life, increased empathy towards complying with dietary restrictions, and increased student group cohesiveness
Patnaik et al. (2020)	**Quantitative**: Validated Likert scale-based, voluntary survey, assessing competency of medical students in 25 nutrition topics, their own dietary consumption habits (including food groups, diets and a validated Med-diet score), and beliefs regarding value of nutrition counseling. Analysed using propensity score–weighted logistic regression models with reported odds ratios and two-tailed *p*-values	Within the multi-site trial using CHOP, University of Texas Health Sciences Center students were significantly more likely to strongly agree nutrition assessment should be routine clinical practice and that providers can improve patients’ health with nutrition education but displayed non-significant results in 12 of the 25 nutrition competency topics
Razavi et al. (2020)	**Quantitative**: Validated Likert scale-based, voluntary survey assessing the competency of medical students in 25 nutrition topics, students' dietary consumption habits (including food groups, diets and a validated Med-diet score), and beliefs regarding the value of nutrition counseling. Analysed using propensity score–weighted logistic regression models with reported odds ratios and two-tailed *p*-values	CHOP participants were at least twice as likely to adhere to Med-diet guidelines involving monounsaturated fats (*P* = 0·009), fruit (*P* = 0·019), vegetables (*P* < 0·001) and legumes (*P* < 0·001), and six times as likely to master Mediterranean diet principles competency
Rothman et al. (2020)	**Quantitative**: Pre- and post-course surveys of students’ self-perceived nutrition knowledge, confidence in counseling patients in management of diet-associated disease, and personal dietary/food choices. Analysed using calculations of means, standard deviations and two-tailed paired t-tests **Qualitative**: Open-ended questions for feedback about the course	The culinary medicine course for final year students, significantly improved competencies in all aspects of nutrition counseling. No significant improvements found in self-perceived value of nutrition counseling and mixed results reported for personal dietary habits
Lieffers et al. (2021)	**Qualitative**: open ended, post- workshop survey focused on workshop evaluation, and reflection on learning	Students enjoyed the workshops for several reasons including the topic itself, cooking, free food, and the chance for interprofessional networking and socialization. Many students self-reported increased knowledge in the workshop topics
Magallanes et al. (2021)	**Quantitative**: pre- and post- intervention Likert scale surveys of confidence in patient nutritional counseling, familiarity with the Mediterranean diet, knowledge of a dietician's role on a care team, personal beliefs on how physicians' nutrition can impact patient outcomes, cooking skills, and perception of the importance of nutritional education in medical programs; chi-squared analysis and calculated odds ratios reported with Yates correction	At baseline most students (92%) strongly agreed or agreed that counseling patients on food choices is essential, but only 29% felt confident in their ability to counsel patients about food choice. and just over half (54%) were familiar with the Mediterranean diet. Post intervention 92% of students felt comfortable discussing nutrition with patients (OR = 26·8), 97% felt familiar with the Mediterranean diet (OR = 25·59) and 93% were confident they understood the role of a dietitian (OR = 23·3). Post intervention, there was a significant increase in the number of students feeling confident in the kitchen (OR = 32·6), and a significant decrease in students who believed healthy eating is expensive or time consuming (OR = 0·43). reporting to think healthy eating is expensive. At baseline students also reported poor understanding of the role of a registered dietician and only 54% of students were familiar with the Mediterranean diet. After the intervention, 92% of students reported feeling confident in counseling patients on nutrition and 93% of students reported to understand the role of a registered dietician. After the intervention, there was a significant increase in the number of students feeling confident in kitchen skills, and a significant decrease in students reporting to think healthy eating is expensive
Asano et al. (2021)	**Quantitative:** Pre- and post-course surveys including Likert scales Qualitative: one open-ended response	Compared to the pre-course survey, students who responded “strongly agree” in questions related to nutrition counseling in the post-course survey were 26·5 to 31·3% higher (*p* < 0·05)In the post-course survey (*n* = 34), 33 students responded to either “strongly agree” (*n* = 25, 73·5%) or “agree” (*n* = 8, 23·5%) that the course increased their knowledge of nutrition
Leggett et al. 2021	**Quantitative:** post-intervention survey; questions assessed participants’ confidence in preparing meals, satisfaction with organization and process of the short experimental course, likelihood of participating or recommending proposed elective to a friend	Almost all participants gave positive ratings to the workshop for improving cooking skills and the quality of the sessions. Most importantly, participants were willing to take the elective if offered, and were overall very likely to recommend the elective to other students. Participants were less certain about the applicability of the elective towards counseling future patients
Poulton & Antono (2021)	**Qualitative:** Pre- and post- surveys of self-perceived confidence in lifestyle medicine topics and on the course itself	In the pre-course survey only 29% of students agreed that they were comfortable counseling patients on nutrition. In the post course survey, 95% of students felt comfortable counseling patients on nutrition
Hashimi et al. (2020)	**Quantitative:** Students completed an anonymous postexperience survey utilizing a Likert‑type rating scale followed by **Qualitative:** free‑text response questions	Most students (91·3%) who completed the post-experience survey, “strongly agreed” or “agreed” they would recommend the experience to other medical students. Most students (60·5%) reported learning something about nutrition and 42·2% reported learning something about cooking. Of the third-year participants (*n* = 19), 94·7% of them reported learning something about nutrition education, and 84.2% of them reported learning something about the social determinants of health. A common theme from the free text responses was the need for more robust education in nutrition science in the initial year of the program
Vanderpool et al. (2020)	**Quantitative**: The "Cooking with a Chef" survey was utilized which consists of 8 sections including 1 index, 6 scales, and 1 knowledge test. A "Culinary medicine curriculum delivery observation checklist" was utilized to measure student engagement, and a "Culinary Medicine Curriculum Delivery Participant Feedback Questionnaire" was utilized for feedback **Qualitative**: An open ended "curriculum focus group" was held post-course	Students demonstrated significant improvements (*P* < ·05) in all assessment measures. Significant improvements identified in cooking confidence and self-efficacy from pre-course survey to post-course survey
D'adamo et al. (2021)	**Quantitative:** Pre-/post session questionnaires assessed nutrition knowledge, skills, and attitudes as well as nutritional counseling confidence scored on a 5-point Likert-type scale. Paired t-tests estimated mean differences in outcomes pre- and post-training **Qualitative:** open-ended qualitative questions were administered to all participants and data were subjected to thematic analysis. Two additional survey questions reflecting on the applicability of the culinary medicine training to patient care and self-care, with open-ended options to elaborate, were asked (only at) in the post training assessment	Participants were asked: "How will you offer practical nutrition advice to your future patients?3 key themes emerged: (1) addressing barriers to healthy eating, (2) personalizing dietary recommendations, and (3) providing evidence-based information for different ways of healthy eating. When participants were asked: " “How do you think you will utilize the information you have learned in the culinary medicine session in your own life?” 3 key themes emerged: (1) implementing cost and time efficiency strategies, (2) trying different evidence-based diets, and (3) making practical dietary changes to support personal health
Kaye et al. (2018)	**Qualitative:** Students provided anonymous feedback on the curriculum. Questionnaire specifics not elaborated	The introductory 3 sessions were well received and repeated in subsequent years, with Grocery Shopping on a Budget being the favorite. The experiential nature of the learning, particularly cooking activities and those that provided a patient-level experience, and a focus on health habits were the favorite aspects of the program. Many commented that they enjoyed activities and spending time with their peers outside of the medical classroom. Overall, the curriculum was well received with students desiring to continue activities beyond the first year
Flynn et al. (2019)	**Quantitative:** pre- and post- assessments including a nutrition quiz designed to assess simple nutrition themes that could be useful in providing patients dietary advice, and a 1-page questionnaire assessing current eating behaviours	Participants significantly increased (*P* < 0·000) knowledge of nutrition, assessed by the 100-point nutrition quiz from pre-course to post-course. At the 2-month follow-up, one-third of participants reported spending less on groceries and an overwhelming number of participants reported to still use the recipes learned in the program
Kumra et al. (2021)	**Quantitative:** Participants completed a voluntary questionnaire which included a 5-point Likert scale (pre-course 23-items with 3 demographic questions; post- course included an additional19 follow-up questions) addressing participants’ attitudes and confidence in providing nutritional counseling to patients and the impact of nutritional counseling and culinary education on patient health outcomes	Significant improvements were reported in self-reported understanding of the principles of motivational interviewing (*P* = 0·002), confidence in ability to use motivational interviewing (*P* < ·001), and intention to use motivational interviewing in their practice (*P* = 0·03). A significant increase (*P* = ·008) was also reported in confidence in ability to come up with nutrition related changes to improve health ( and intention to discuss nutrition related changes with patients (*P* = 0·006)
Musick et al. (2020)	**Quantitative:** faculty assessed each student’s attendance, preparation and level of participation in all required activities, with students earning 0 points (absent or disruptive group behavior), 1 point (no, minimal or inappropriate participation) or 2 points (effective participation). Students also submitted reflective essays (between 400 and 500 words) that were graded by a "Team Action Group" composed of 7 faculty members with students earning from 0 to 3 points for their essays. Students from all 3 disciplines used a Likert-type scale to provide feedback for the course, ranking items that reflected how the course prepared them for interprofessional learning and patient interactions surrounding nutritional counseling	While generally received positively by students, this new curricular track was very labor- intensive and not particularly impactful in terms of orienting students to the demands of clinical patient care teams. Students rated their experiences in learning about nutrition favorably (3·78 on a five-point Likert scale) and many students expressed appreciation for this content anecdotally

*CHOP* Cooking Through Health Optimisation, *OR* Odds ratio, *CI* Confidence interval, *P* = *P* value, *t* = T-test value, *Med-diet* Mediterranean diet, *PBL* Problem-based learning, *RI* Rhode Island

### Quality appraisal

The methodological quality and reporting of each study was assessed independently, in duplicate using the CASP tool, version 2020 [11]. CASP has specific appraisal checklists designed for different study designs, both within the study and across studies, making it an appropriate tool for this scoping review. Agreement was reached on all of the appraisal items. With regards to satisfying each CASP criteria, papers were assigned a score of 0 for ‘yes’, 1 for ‘can’t tell’ and 2 for ‘no’. The CASP scores were a sum of the individual criterion scores for each paper. Where scores differed, discrepancies were resolved through team discussions.

## Results

A total of 2289 articles were screened by their title and abstract. 2249 articles were identified through database searches using the culinary medicine search string created for each database, 168 articles found from these databases were removed by Covidence as duplicates. 208 articles were identified through ancestry searching and were screened by their title and abstract but none were found to be suitable to follow up for full-text review. A total of 54 articles were reviewed as a full-text. Of these articles, 24 met the criteria and were included in the scoping review.

Reasons for excluding articles reviewed as full-text included wrong study design (*n*= 13) [4, 12–23], wrong population (*n*= 5) [24–28], a failure to report any outcome measures (*n*= 3) [9, 29, 30], wrong intervention (*n*= 3) [26, 31, 32], and five studies were found to be duplicates of an included paper or had used the same data (*n*= 5) [33–37]. Amongst the 24 included studies, there were six qualitative studies [38–43], four cross-sectional studies [34, 44–46]; seven case studies [47–53], and seven mixed-methods study [35, 54–59].

One study took place in Canada [43], while the other 23 studies took place in the United States of America (USA). The number of participants ranged from four [38] to 4215 [46]. Twenty studies involved medical students only [34, 35, 38–42, 44–46, 48, 49, 51, 52, 54–59], nine of which evaluated a single year cohort of students rather than a mixed cohort between years one through four of their medical program [38, 40, 42, 52, 55–59]. Two studies involved medical students and medical registrars [45, 47], and two also included nutrition students [43, 47]. Only one of the studies did not include a collaborative cooking session [41] and 18 included both didactic and collaborative cooking sessions [34, 38–40, 42–48, 51, 54–59]. A variety of other delivery methods were used amongst the studies including: case-based learning in groups (*n*= 12) [38–40, 43–47, 53, 56, 57, 59]; pre-course preparations in the form of pre-readings, videos, and assignments (*n*= 15) [34, 35, 38–40, 42, 44–48, 55–57, 59]; pre-session quizzes (*n* = 7) [34, 38, 43, 48, 56, 57, 59] and after-class assignments and homework (*n*= 9) [38–40, 42, 48, 55–57, 59].

Twelve studies reported the study population demographics, with participants from varying ethnicities and nutritional backgrounds [34, 44–46, 48–52, 56–58]. The recruitment process in the studies typically involved an open elective application (*n*= 6) [42, 49, 50, 55, 56, 59], or voluntary enrolment into the course (*n*= 8) [38, 39, 43, 44, 48, 51, 52, 54].

All but three studies [41, 47, 53] had an underpinning objective of using culinary medicine approaches to improve medical students’ nutrition knowledge and counseling in a clinical environment to support chronic disease prevention and management (*n*= 21) [34, 35, 38–40, 42–46, 48–52, 54–59]. Studies assessed medical students’ culinary skills (*n*= 4) [52, 54, 56, 59], nutrition attitudes (*n*= 12) [34, 35, 39, 44, 48, 50, 52, 54, 56–59], and provision of nutrition counseling to patients (*n*= 10) [35, 39, 44, 50–52, 55–58]. Studies also assessed student collaboration amongst other populations, such as people in the community (*n* = 4) [38, 44, 56, 57] and other healthcare professionals, including allied health and practicing physicians (*n*= 10) [39, 40, 43, 47, 50, 53–57]. Nine studies made direct comparisons between practical nutrition education in the form of a culinary medicine program against traditional nutritional education in the form of solely didactic teaching [39, 40, 44–48, 51, 54].

Fifteen studies reported statistically significant improvements in outcomes and were therefore considered as effective [34, 44–50, 52, 54–59]. Students’ improvement in nutritional attitudes were reported in pre- and post-course surveys, nine of which achieved statistically significant improvements [34, 44, 48, 52, 54, 56, 59]; four identified statistically significant improvement in culinary skills [52, 54, 56, 59], and 10 identified changes in competency providing nutritional counseling [35, 39, 44, 48, 50, 52, 55–58], eight of which were statistically significant [44, 48, 50, 52, 55–58]. Pre- and post-course surveys from 11 of the included studies identified changes in personal health behaviors [34, 35, 39, 44, 49, 50, 52, 54, 56, 57, 59] and two studies reported student improvements in their ability to identify food by visual inspection [54, 57]. Eleven of the 24 studies failed to mention any negative or non-significant outcomes within their results [35, 38, 39, 41–43, 46–48, 54, 59].

Five studies utilised interventions that were adopted by multiple faculties [34, 44–46, 52]. The interventions were implemented within a number of different settings. The most common settings were teaching kitchens (*n* = 13) [34, 42–48, 52, 54, 56, 57, 59], community kitchens (*n* = 8) [38, 41, 42, 44, 50, 53, 56, 57] and off-site kitchens (*n*= 10) [35, 38–40, 44, 46, 50, 51, 55, 58]. Only eight studies explicitly stated that the interventions met or exceeded the recommendation that US medical education include 25 h in nutrition education [34, 42, 45–48, 53, 59].

The curriculum offered to students in each study varied by session layout, duration, type of instructor/instructors, and whether courses were offered as an elective (*n* = 21) [34, 35, 38, 39, 41–52, 54–57, 59] or non-elective course (*n*= 3) [40, 53, 58]. Half of the interventions were based on an established program, CHOP from Tulane University (*n*= 6) [34, 38, 44–47], and *Health Meets Food* (*n*= 1) [52]. Others used an original program (*n* = 14) [39–43, 49–51, 53–58] or a modified version of a known curriculum (*n* = 3) [35, 48, 59] Seven studies reported modifications in their curriculum between cohorts with all changes made to address student feedback [40–42, 53, 55–57]. Thirteen studies had chef instructors [38, 39, 43, 45, 47, 48, 51, 53–57, 59], nine studies included physicians as instructors [39, 46–48, 50, 52, 53, 55, 57]; six studies included instructors from the school of medicine faculty [38–43, 46–48, 53, 56, 58, 59], and nine studies from the school of nutrition faculty [39, 41, 43, 45–47, 51, 53, 55]; five studies involved instruction from hospital dietitians [47, 50, 52, 54, 57]; and four studies included medical students teaching peers [42, 43, 54, 57]. Three studies included a service component where medical students taught and/or served members of the community [34, 40, 57]. Of all the included papers, two reported observations from the facilitators on the programs themselves following completion of the course [38, 53].

None of the included papers explicitly reported the complete cost of running these programs. In only one paper, the cost of cooking materials, in addition to the license for Tulane’s culinary medicine curriculum were reported [38].

### Quality appraisal

The mean CASP score was 9.56 out of a possible 20 points for qualitative studies (median = 10, range = 7–13) and 4.13 out of a possible 24 (Median = 4, range = 0–7) for cohort studies. The most frequent items that were not achieved amongst the cohort studies included that the authors did not identify all important confounding factors (9/15) [39, 48–53, 58, 59] and the cohort study was not recruited in an acceptable way (7/15) [39, 45–47, 50, 53, 54] Amongst the cohort studies, it was also found that the follow up of subjects was not sufficiently complete (6/15 studies) [39, 44–47, 54], with the most common reasons including unclear reporting or high attrition rates of participants. Many of the cohort studies also lacked an acceptable recruitment method (7/15 studies) [39, 45–47, 50, 53, 54], with the most common reasons including unclear reporting, lack of inclusion/exclusion criteria and the reliance of convenience sampling biased towards students with a ‘voluntary’ or ‘elective’ interest. Half of the studies failed to report attrition rates (*n* = 12) [34, 38–40, 42–46, 53, 55, 59] and only two of these studies reported reasons why the participants failed to complete the course [52, 54].

The overall poorer quality of the qualitative studies was related to the frequent absence of several CASP items in the studies. The relationship between the researchers and participants was poorly reported in all nine qualitative studies, most frequently due to lack of reporting of any such considerations in the methodology [35, 38, 40–43, 55–57]. In addition, all included papers displayed poor consideration of ethical issues, either due to failure to mention any ethical considerations or having their ethical approval waived by the institution [56]. All included papers also had an insufficiently rigorous analysis of data, most commonly due to a lack of data presented and analysis performed. The findings of the CASP quality appraisal, with reasons for negative scoring per criterion, are described in Tables 3 and 4.

**Table 3:** Qualitative study appraisal

Criterion	Dreibelbis & George, 2017	Ring et al., 2019	Pang et al., 2019	Hennrikus et al., 2020	Rothman et al., 2020	Lieffers et al., 2021	Poulton & Antono, 2021	Hashimi et al., 2020	Kaye et al., 2018
Was there a clear statement of the aims of the research?	Yes	Yes	Yes	Yes	No ^a^	Yes	No ^a^	Yes	Yes
Is a qualitative methodology appropriate?	Can't Tell ^b^	No ^c/d^	Yes	Can’t Tell ^b^	Yes	Yes	Yes	Yes	Yes
Worth Continuing?	Yes	No ^c/d^	Yes	Yes	Yes	Yes	Yes	Yes	Yes
Was the research design appropriate to address the aims of the research?	No ^a^	No ^c/d^	Yes	Yes	No ^e^	No ^a^	Can’t Tell ^a^	No ^c/f^	Yes
Was the recruitment strategy appropriate to the aims of the research?	No ^a^	No ^d^	No ^c^	Yes	Yes	No ^a^	Can’t Tell ^a^	No ^a/d^	Yes
Was the data collected in a way that addressed the research issue?	Can't Tell ^a/b^	Yes	Yes	Can't Tell ^b^	No ^g^	Can’t Tell ^a^	Yes	Can’t Tell ^c^	Yes
Has the relationship between researcher and participants been adequately considered?	No ^a/f^	No ^a^	No ^a^	No ^a^	No ^a^	No ^a^	Can’t Tell ^a^	Can’t Tell ^a^	No ^a^
Have ethical issues been taken into consideration?	Can't Tell ^a^	No ^a^	No ^a^	No ^a^	No ^a^	Can’t Tell ^a^	Can’t Tell ^a^	No ^f/a^	Yes
Was the data analysis sufficiently rigorous?	No ^a/b/f/g^	No	No ^b/f^	No ^b/f^	Can't Tell ^g^	No ^g^	Can’t Tell ^f^	No ^f/a^	No ^f^
Is there a clear statement of findings?	No ^g^	Yes	No ^f^	Yes	Yes	No ^g^	No ^f/g^	Yes	No ^a^
**Uncertainty Score**	13	12	10	8	11	7	9	10	6

Reasons: ^a^Not reported, ^b^Selection bias/Convenience sampling/Elective course, ^c^Poorly designed surveys, ^d^Inadequate controlling for multiple confounders, ^e^High attrition rate, ^f^Not performed, ^g^Poor overall quality of study, ^h^Low sample size

**Table 4:** Cohort study appraisal

**Criterion**	**Monlezun et al., 2015**	**Jaroudi et al., 2018**	**Monlezun et al., 2018**	**Lawrence et al., 2019**	**Hauser et al., 2019**	**Patnaik et al., 2020**	**Razavi et al., 2020**	
Did the study address a clearly focused issue?	Yes	Yes ^a^	Yes	Yes	Yes	Yes	Yes	
Was the cohort recruited in an acceptable way?	Yes	Can't Tell ^b^	Yes	Can't Tell ^a^	Can't Tell ^a^	Can't Tell ^b^	Can't Tell ^b^	
Worth Continuing?	Yes	Yes	Yes	Yes	Yes	Yes	Yes	
Was the exposure accurately measured to minimise bias?	Yes	Yes	Yes	Yes	Yes	Can't Tell ^b^	Can't Tell ^b^	
Was the outcome accurately measured to minimise bias?	Yes	Yes	Yes	Yes	Can't Tell ^a^	Can't Tell ^b^	Can't Tell ^c^	
Have the authors identified all important confounding factors?	Yes	Yes	Yes	Yes	Can't Tell ^a^	Yes	Yes	
Have they taken account of the confounding factors in the design and/or analysis?	Yes	Can't Tell ^d^	Yes	Can't Tell ^c/e^	Can't Tell ^a^	Yes	Yes	
Was the follow up of subjects complete enough?	Yes	No	Can't Tell ^a^	No	Can't Tell ^a^	Can't Tell ^f^	Can't Tell ^f^	
Was the follow up of subjects long enough?	Yes	Can't Tell ^a^	Can't Tell ^a^	Can't Tell ^a^	Yes	Can't Tell ^f^	Can't Tell ^f^	
Do you believe the results?	Yes	Yes	Yes	Yes	Can't Tell ^g/h^	Yes	Yes	
Can the results be applied to the local population?	Yes	No ^h/g^	Yes	No ^h/g^	Can't Tell ^g/h^	Yes	Yes	
Do the results of this study fit with other available evidence?	Yes	Yes	Yes	Yes	Yes	Yes	Yes	
**Uncertainty Score**	0	7	2	7	7	5	5	
**Criterion**	**Magallanes et al., 2021**	**Leggett et al., 2021**	**Asano et al., 2021**	**D’adamo et al., 2021**	**Flynn et al., 2019**	**Musick et al., 2020**	**Kumra et al., 2021**	**Vanderpool et al., 2020**
Did the study address a clearly focused issue?	Yes	Yes	Yes	Yes	Yes	Yes	Yes	Yes
Was the cohort recruited in an acceptable way?	Yes	Yes	Yes	Yes	Yes	Can't Tell ^b^	Can't Tell ^b^	Yes
Worth Continuing?	Yes	Yes	Yes	Yes	Yes	Yes	Yes	Yes
Was the exposure accurately measured to minimise bias?	Yes	Yes	Yes	Yes	Yes	Can't Tell ^b^	Yes	Yes
Was the outcome accurately measured to minimise bias?	Yes	Can’t Tell ^a^	Yes	Yes	Yes	Can't Tell ^a^	Yes	Yes
Have the authors identified all important confounding factors?	Can’t Tell ^a^	Can’t Tell ^a^	Can’t Tell ^d^	Can’t Tell ^a^	No ^d^	No ^f^	No ^b/f^	No ^b^
Have they taken account of the confounding factors in the design and/or analysis?	No ^a^	Yes	Can’t Tell ^a^	Yes	No ^f^	Can’t Tell ^a^	Can’t Tell ^a^	Yes
Was the follow up of subjects complete enough?	Yes	Yes	Yes	Yes	Yes	Yes	Yes	Yes
Was the follow up of subjects long enough?	Yes	Yes	Yes	Yes	Yes	Yes	Yes	Yes
Do you believe the results?	Yes	Yes	Yes	Yes	Yes	Yes	Yes	Yes
Can the results be applied to the local population?	Yes	Can’t Tell ^c^	Yes	Yes	Yes	No ^i^	Yes	No ^h^
Do the results of this study fit with other available evidence?	Yes	Yes	Yes	Yes	Yes	Can’t Tell ^g^	Yes	Yes
**Uncertainty Score**	3	3	1	1	4	9	4	4

Reasons: ^a^Not reported, ^b^Selection bias/Convenience sampling/Elective course, ^c^Poorly designed surveys, ^d^Inadequate controlling for multiple confounders, ^e^High attrition rate, ^f^Not performed, ^g^Poor overall quality of study, ^h^Low sample size, ^i^Population difficult to reproduce

## Discussion

This study is one of the first reviews, along with Patel & Kassam 2021 and Asher et al. 2022, to advance understanding of current opportunities and obstacles for culinary medicine within the context of medical, or other health care professional education, by examining the impact of culinary medicine in medical programs using a systematic approach [4, 18]. In recent years, a clear interest in the use of culinary medicine as an education tool has been demonstrated in the literature. Between the initial search conducted in June 2020, and a second identical search conducted in April 2022, 11 new papers were published that sought to evaluate culinary medicine teaching as a viable option to teach, or support the teaching of, nutrition in medical school [35, 41, 43, 48–53, 58, 59]. Many pilot and trial implementations indicate promising efficacy towards improving medical students’ nutritional knowledge, skills and attitudes [34, 44, 48, 52, 54, 56, 57, 59].

The majority of included studies were published in the last half decade and achieved some statistically significant outcomes that promote a hands-on method of nutritional education over traditional didactic methods to educate students to help combat chronic disease as future physicians. Current literature is in line with the growing interest in culinary medicine programs, citing its emergence to the inadequacies of conventional education in combatting the rising burden of chronic disease in the healthcare sector [6, 32]. This review demonstrates clear interest in the use of hands-on culinary medicine initiatives, as an educational tool and replacement for the standard curriculum of medical students. Given the relative recency of the published research, this review provides incentive for medical educators to continue to innovate and implement culinary medicine initiatives into medical education. Papers published before 2020 generally reflect poorer quality of studies, large ranges in sample size with varied results, and choice of study designs (mainly qualitative, pilot-studies). In more recent publications, the pilot programs have demonstrated greater quality and have mostly reported quantitative data with a case study design, or a mixed methods study design, allowing for both quantitative assessment of student improvement pre- and post- intervention, as well as qualitative data obtained in the form of free-form evaluation from participants after the completion of the intervention [35, 48–53, 58, 59].

All included papers in this review were published in North America, which may suggest that the increasing interest in culinary medicine appears limited in geographical footprint. However, this focus on North America highlights an opportunity for broader international involvement to legitimise the culinary medicine curriculum as a relevant approach for nutrition training in medical education. It also brings to light the important consideration of cultural relevancy and potential need for modification when international protocols eventuate.

Eighteen of the culinary medicine programs included in our review had a didactic component, with 14 programs utilising pre-coursework and 12 programs also utilising a case-based group learning format in addition to the hands-on component. Problem based learning, lecture sessions (live or recorded), and pre- coursework are commonplace methods of delivery in medical school curricula and have been proven as an effective means for educating medical students [60, 61]. Hence, it seems reasonable that these delivery methods would be adequate for delivery of culinary medicine teaching in combination with hands-on kitchen sessions, providing further merit to the feasibility and implementation of hands-on culinary medicine programs. However, there were also several variations both in the delivery method (i.e., pre-session quizzes, homework, community involvement, multidisciplinary faculty), setting (i.e., faculty resources, community resources), and aspects of culinary medicine taught (knowledge, skills, attitudes). These variations in culinary medicine programs have been well documented in the literature, leading to a myriad of considerations necessary to planning an effective intervention [14]. Given that the few studies which acquired data across multiple universities as part of their curriculum [34, 44, 45, 55] all sourced their curriculum from Tulane University’s Cooking for Health Optimization with Patients (CHOP) curriculum, there is an evident scarcity of a globally accepted standardised curricula. This further exemplifies the need to provide clear objectives to guide future interventional studies and for medical schools interested in utilising culinary medicine to integrate the key principles adopted by schools, with established curricula, such as those from Tulane University [19].

None of the included studies reported the total cost of running the program or performed any cost analysis. Only one study reported the costs of cooking materials, and license for Tulane’s culinary medicine curriculum [38]. While previous studies have attempted to address this gap in the literature by directly inviting program directors to comment on program costs/funding among other aspects, the majority of directors elected to provide approximate round figures without further cost breakdown, or simply elect to classify program cost as confidential [7]. Regardless of cost–benefit or cost-effectiveness analyses, lack of reporting for simple expenditures in most interventions makes it challenging to assess whether culinary medicine initiatives can be integrated into existing medical programs as the costs associated with personnel, facilities and equipment/consumables may vary significantly between programs and location. While this is likely due to most studies being pilot studies, a medical qualification is already among the most expensive university programs available to students worldwide, and costs continue to increase at the expense of accessibility [5]. Given the scarcity of outcome data and the intrinsic difficulty in comparing learning outcomes in the context of future benefit to patients combined with the ethical and epistemological difficulty in performing a cost benefit/effectiveness analysis in the setting of medical education, it is recommended that at minimum future studies provide data relating to the monetary costs; allowing the possibility of cost–benefit meta-analyses to be performed once higher quality data related to outcomes are published [5].

In studies which assessed students’ nutrition competencies, significant improvements were reported when compared to traditional teaching methods. The key difference was that culinary medicine curricula offers hands-on practical components and case-based learning as shown in Table 1. These practical culinary components are analogous to lab-based experiential learning, allowing students to apply theoretical knowledge to simulate real-world patient cases and to “learn by doing”,—an effective method for clinical knowledge translation [62, 63]. Other possible reasons for the improved competency outcomes arise from multidisciplinary faculty and community service components that together helped to increase students’ understanding in applying practical nutrition and dietary advice in a community context [64]. Given that physicians play a key role in advising patients in nutrition, enhanced nutrition counseling skills will be effective for improving long term health outcomes for patients struggling with diet, weight loss, diabetes, and other chronic health problems [65]. If increased nutrition counseling competencies gained through culinary medicine programs can be maintained into vocational practice, future doctors will be better equipped to address the incoming burden that chronic diseases poses for healthcare systems [66].

While it is promising that increased confidence in culinary skills and nutrition knowledge were demonstrated through participation in culinary medicine programs, none of the included studies sought longer-term follow-up with participants, limiting current understanding of the enduring effects of these interventions. This is an important consideration. Four out of 9 of the qualitative studies included in this review had cross-sectional methodologies, which under the *Levels of Evidence Framework*, form the lowest level evidence in establishing causation, but provide a basis for future study designs to incorporate stronger forms of evidence [67]. Since the ultimate objective for culinary medicine programs is, positive clinical practice outcomes, it would be ideal for future studies to provide more robust evidence when investigating the impact of nutritional competencies gained within a culinary medicine program correlating or translating to better nutrition counseling in practice. Longitudinal studies would be required that track participants post-graduation and involve suitable controls for comparison, which could be a challenging aspect of study design.

The findings of surveys on students’ attitudes towards nutrition counseling showed mixed results. Two studies identified negative findings, which while initially surprising, were partially explained by examining the recruitment methods used. In Pang et al. (2019) suitable participants were assessed by application essays, allowing course conveners to select participants based on perceived interest, resulting in potential selection bias. Furthermore, the majority of studies reviewed were electives. Together, these two factors indicate strong selection bias, that of course conveyors selections and participants’ personal inclinations, and resulted in high positive pre-course scores on attitudes towards nutrition and nutrition counseling and statistically non-significant results. These negative findings when analysed in tandem with post-course improvements in nutrition counseling competencies suggest that students’ individual attitudes towards nutrition is not the largest factor contributing to their lack of nutritional knowledge to effectively counsel patients about diet. Nonetheless, future primary studies assessing culinary medicine programs should attempt to mitigate inherent selection bias and confounding results. Mitigation could potentially be achieved by implementing culinary medicine as a non-selective component with randomised participants and consequent non-participants as controls.

### Strengths and limitations of this review

The included papers in this study were collated from multiple databases through keyword search strings to yield results relevant to the research question, which has the potential be too narrow in scope. Screening of 2289 relevant papers was performed manually through Covidence after establishing 98% interrater reliability for an initial 50 results. Whilst interrater reliability was high, the potential for exclusion of relevant papers for final review does exist to some extent. Given that culinary medicine is a relatively recent development in the context of medical pedagogy, all the primary research findings were the results from pilots and on-going trials with limited longitudinal data and differing methodologies. Despite the best efforts to make data comparable by overlaying a systematic approach on qualifying studies, interpretation of results necessarily requires extrapolation to draw relevant pedagogical and clinical conclusions. These subjective effects were mitigated, in-part, by citing peer-reviewed papers that supported our analysis. Another consequence of disparate measurement methods between existing studies is the inability to apply quantitative statistical methods in a meaningful manner, such as regression analysis and heterogenicity calculations. Specific challenges to overcome this limitation include accounting for duplicity of results for papers involved in a same trial and determining which outcome performance measures to use. Moreover, publication biases could obfuscate true effects of culinary medicine interventions if significant results are published more often than non-significant findings. Furthermore, as many of the results in these trials are likely to have an impact on the continued institutional support of these pilot programs, there is a risk of confirmation biases from investigators. Although study quality was not used to exclude papers used for our review, we have modified the CASP protocol to obtain a numerical rank of study quality (Tables 3 and 4). Several steps within the CASP protocol requires assessors’ judgement to determine if a criterion has been met and this process is inherently subjective and may vary between individuals.

While our search was performed in English, which is inherently biased towards the selection of papers produced in Anglophone countries or papers with English translations embedded, we recognise that there may be relevant papers in other languages that have been unintentionally excluded. Tangential to this issue is the unintended isolation of papers reviewed to only the United States of America and Canada, which raises the question of generalisability of our findings towards medical teaching institutions in other countries.

Despite these limitations, this is one of the first scoping reviews of the literature on culinary medicine programs that detail existing culinary medicine program components, methods and results. Through our analysis, future investigations will be able to rely on a consolidated paper to determine appropriate study designs, types of data collection and analytical methodologies.

## Conclusions

This paper identified a small but notable body of literature describing culinary medicine programs that have been implemented in medical schools and analysed their findings. Our results indicate that culinary medicine programs are a good initiative to pursue and can be delivered in a hands-on way that provides potential future clinical benefits for students and patients. Culinary medicine programs appear to be a feasible replacement for traditional didactic nutrition education and may be more effective than traditional didactic methods at improving student competency. Yet, despite growing interest in establishing culinary medicine curricula and positive pilot and trial data, there is still a lack of strong evidence to claim that culinary medicine programs are superior to traditional nutrition education in medical school. A lack of standardization between culinary medicine pilot studies further hinders comparability and the ability to extrapolate benefits.

While more research is needed to determine the viability of culinary medicine programs in medical education, the argument for implementation can be improved if future culinary medicine studies obtain stronger evidence and maintain consistent objectives and methodologies as established in current literature. Namely, maintaining consistency with competency scales, teaching staff mix, delivery methods and qualitative analytical methods. Future studies should also aim to report on program cost and provide long term follow up of participants.

## Supplementary Information

**Additional file 1.**

**Additional file 2.**

## Data Availability

The datasets generated during and/or analysed during the current study are presented in Tables 1 and 2 in the manuscript. An example of data charting is included in Table 1 in the supplementary material provided. Raw data sets are available from the corresponding author on reasonable request.

## References

[1] Burnett R, Kyu HH, Thomas BA, Abubakar I, Abu-Rmeileh NME, Albittar MI, Aleman AV, Alsharif U, Anderson BO, Arsenijevic VSA (2015). Global, regional, and national comparative risk assessment of 79 behavioural, environmental and occupational, and metabolic risks or clusters of risks in 188 countries, 1990–2013: a systematic analysis for the Global Burden of Disease Study 2013. The Lancet.

[2] Crowley J, Ball L, Hiddink GJ (2019). Nutrition in medical education: a systematic review. Lancet Planet. Health.

[3] Steeves JA, Liu B, Willis G, Lee R, Smith AW (2014). Physicians’ personal beliefs about weight-related care and their associations with care delivery: The U S National Survey of Energy Balance Related Care among Primary Care Physicians. Obes Res Clin Pract.

[4] Asher RC, Shrewsbury VA, Bucher T, Collins CE (2022). Culinary medicine and culinary nutrition education for individuals with the capacity to influence health related behaviour change: a scoping review. J Hum Nutr Diet.

[5] Irl BH, Evert A, Fleming A, Gaudiani LM, Guggenmos KJ, Kaufer DI, McGill JB, Verderese CA, Martinez J (2019). Culinary medicine: advancing a framework for healthier eating to improve chronic disease management and prevention. Clin Ther.

[6] La Puma J (2016). What is culinary medicine and what does it do?. Popul Health Manag.

[7] Coppoolse HL, Seidell JC, Dijkstra SC (2020). Impact of nutrition education on nutritional knowledge and intentions towards nutritional counselling in Dutch medical students: an intervention study. BMJ Open.

[8] Monlezun DJ, Kasprowicz E, Tosh KW, Nix J, Urday P, Tice D, Sarris L, Harlan TS (2015). Medical school-based teaching kitchen improves HbA1c, blood pressure, and cholesterol for patients with type 2 diabetes: results from a novel randomized controlled trial. Diabetes Res Clin Pract.

[9] Hauser ME, Nordgren JR, Adam M, Gardner CD, Rydel T, Bever AM, Steinberg E (2020). The first, comprehensive, open-source culinary medicine curriculum for health professional training programs: a global reach. Am J Lifestyle Med.

[10] Page MJ, McKenzie JE, Bossuyt PM, Boutron I, Hoffmann TC, Mulrow CD, Shamseer L, Tetzlaff JM, Akl EA, Brennan SE (2021). The PRISMA 2020 statement: An updated guideline for reporting systematic reviews. Int J Surg.

[11] Critical Appraisal Skills Programme UK. (n.d.). CASP checklists. Retrieved from https://casp-uk.net/casp-tools-checklists/.

[12] Adams KM, Kohlmeier M, Powell M, Zeisel SH (2010). Nutrition in medicine: nutrition education for medical students and residents. SAGE Publications.

[13] Barkoukis H, Swain J, Rogers C, Harris SR (2019). Culinary medicine and the registered dietitian nutritionist: time for a leadership role. J Acad Nutr Diet.

[14] Hauser ME. Culinary Medicine Basics and Applications in Medical Education in the United States. Nestle Nutr Inst Workshop Ser. 2019;92:161–70. 10.1159/000499559 31779011

[15] Leong B, Ren D, Monlezun D, Ly D, Sarris L, Harlan TS (2014). Teaching third and fourth year medical students how to cook: an innovative approach to training students in lifestyle modification for chronic disease management. Med Sci Educ.

[16] Merlo G, Tollefson M, Dacey M, Lenz T, Luchsinger M, Muscato D, Frates EP (2020). Laying an Early Foundation: Lifestyle Medicine Pre-Professional Education (LMPP) Member Interest Group. SAGE Publications..

[17] Mondala MM, Sannidhi D (2019). Catalysts for change: accelerating the lifestyle medicine movement through professionals in training. SAGE Publications..

[18] Patel P, Kassam S: Evaluating nutrition education interventions for medical students: A rapid review. Journal of Human Nutrition and Dietetics 2021 10.1111/jhn.12972 PMC9546301 34842308

[19] Polak R, Phillips EM, Nordgren J, La Puma J, La Barba J, Cucuzzella M, Graham R, Harlan T, Burg T, Eisenberg D (2016). Health-related culinary education: a summary of representative emerging programs for health professionals and patients. Glob Adv Health Med.

[20] Rao M, Agarwal P (2021). Culinary medicine: exploring diet with tomorrow’s doctors. Canadian Med Educ J.

[21] Sicker K, Habash D, Hamilton L, Nelson NG, Robertson-Boyd L, Shaikhkhalil AK (2020). Implementing culinary medicine training: collaboratively learning the way forward. J Nutr Educ Behav.

[22] Stiegmann RA, Abreu A, Gardner JE, Hipple JM, Poling PE, Frates EP (2017). Planting the seeds of change: growing lifestyle medicine interest groups with the Donald A. Pegg award. Am J Lifestyle Med.

[23] Williams A, Diffenderfer A, Carlyle K (2020). Hands-on cooking in medical schools: diffusion of a prevention education innovation. Med Sci Educ.

[24] Doxey RS, Krug MF, Tivis R (2021). The lunch conference diet: fostering resident engagement in culinary medicine through a curriculum centered on changes to provided conference food. Am J Lifestyle Med.

[25] Johnston EA, Arcot A, Meengs J, Dreibelbis TD, Kris-Etherton PM, Wiedemer JP (2021). Culinary medicine for family medicine residents. Med Sci Educ.

[26] Olfert MD, Wattick RA, Hagedorn RL (2020). Experiences of multidisciplinary health professionals from a culinary medicine cultural immersion: qualitative analysis. Health Prof Educ.

[27] Santella ME, Hagedorn RL, Wattick RA, Barr ML, Horacek TM, Olfert MD (2020). Learn first, practice second approach to increase health professionals’ nutrition-related knowledge, attitudes and self-efficacy. Int J Food Sci Nutr.

[28] Stauber Z, Razavi AC, Sarris L, Harlan TS, Monlezun DJ (2022). Multisite medical student-led community culinary medicine classes improve patients’ diets: machine learning-augmented propensity score-adjusted fixed effects cohort analysis of 1381 subjects. Am J Lifestyle Med.

[29] Birkhead AG, Foote S, Monlezun DJ, Loyd J, Joo E, Leong B, Sarris L, Harlan TS (2014). Medical student-led community cooking classes: a novel preventive medicine model that’s easy to swallow. Am J Prev Med.

[30] Jackson AA (2001). Human nutrition in medical practice: the training of doctors. Proceedings of the Nutrition Society.

[31] Afaghi A, Haj Agha Mohamadi AA, Ziaee A, Sarchami R (2012). Effect of an integrated case-based nutrition curriculum on medical education at Qazvin University of Medical Sciences. Iran Glob J Health Sci.

[32] Blunt SB, Kafatos A (2019). Clinical nutrition education of doctors and medical students: solving the catch 22. Adv Nutr (Bethesda, Md).

[33] Dreibelbis TD, George DR (2017). Integrating intergenerational mentoring into a culinary medicine curriculum. Med Sci Educ.

[34] Monlezun DJ, Dart L, Vanbeber A, Smith-Barbaro P, Costilla V, Samuel C, Terregino CA, Abali EE, Dollinger B, Baumgartner N (2018). Machine learning-augmented propensity score-adjusted multilevel mixed effects panel analysis of hands-on cooking and nutrition education versus traditional curriculum for medical students as preventive cardiology: multisite cohort study of 3,248 trainees over 5 years. Biomed Res Int.

[35] Poulton G, Antono A (2022). A taste of virtual culinary medicine and lifestyle medicine—an online course for medical students. Am J Lifestyle Med.

[36] Razavi AC, Dyer A, Jones M, Sapin A, Caraballo G, Nace H, Dotson K, Razavi MA, Harlan TS (2020). Achieving dietary sodium recommendations and atherosclerotic cardiovascular disease prevention through culinary medicine education. Nutrients.

[37] Stauber Z, Razavi AC, Sarris L, Harlan TS, Monlezun DJ (2022). Multisite medical student-led community culinary medicine classes improve patients’ diets: machine learning–augmented propensity score-adjusted fixed effects cohort analysis of 1381 subjects. Am J Lifestyle Med.

[38] Dreibelbis TD, George DR (2017). An Intergenerational Teaching Kitchen: Reimagining a Senior Center as a Shared Site for Medical Students and Elders Enrolled in a Culinary Medicine Course. J Intergenerational Relationships.

[39] Hauser ME (2019). A novel culinary medicine course for undergraduate medical education. Am J Lifestyle Med.

[40] Hennrikus EF, Skolka MP, Hennrikus N (2020). Social constructivism in medical school where students become patients with dietary restrictions. Adv Med Educ Pract.

[41] Hashimi H, Boggs K, Harada C (2020). Cooking demonstrations to teach nutrition counseling and social determinants of health. Educ Health (Abingdon).

[42] Kaye S, Pathman J, Skelton JA (2019). Development and implementation of a student-led lifestyle medicine curriculum. Am J Lifestyle Med.

[43] Lieffers J, Wolfson E, Sivapatham G, Lang A, McEwen A, D’Eon M, Henry C (2021). Interprofessional culinary education workshops at the University of Saskatchewan. Can Med Educ J.

[44] Monlezun DJ, Leong B, Joo E, Birkhead AG, Sarris L, Harlan TS (2015). Novel longitudinal and propensity score matched analysis of hands-on cooking and nutrition education versus traditional clinical education among 627 medical students. Adv Prev Med.

[45] Patnaik A, Tran J, McWhorter JW, Burks H, Ngo A, Nguyen TD, Mody A, Moore L, Hoelscher DM, Dyer A (2020). Regional variations in medical trainee diet and nutrition counseling competencies: machine learning-augmented propensity score analysis of a prospective multi-site cohort study. Medical Science Educator.

[46] Razavi AC, Monlezun DJ, Sapin A, Stauber Z, Schradle K, Schlag E, Dyer A, Gagen B, McCormack IG, Akhiwu O (2020). Multisite culinary medicine curriculum is associated with cardioprotective dietary patterns and lifestyle medicine competencies among medical trainees. Am J Lifestyle Med.

[47] Lawrence JC, Knol LL, Clem J (2019). de la O R, Henson CS, Streiffer RH: Integration of Interprofessional Education (IPE) Core Competencies Into Health Care Education: IPE Meets Culinary Medicine. J Nutr Educ Behav.

[48] Asano S, Jasperse AE, Schaper DC, Foster RW, Griffith BN (2021). A culinary medicine elective course incorporating lifestyle medicine for medical students. Med Sci Educ.

[49] Flynn MM, George P, Schiffman FJ (2019). Food is medicine: using a 4-week cooking program of plant-based, olive oil recipes to improve diet and nutrition knowledge in medical students. Med Sci Educ.

[50] Kumra T, Rajagopal S, Johnson K, Garnepudi L, Apfel A, Crocetti M (2021). patient centered medical home cooking: community culinary workshops for multidisciplinary teams. J Prim Care Community Health.

[51] Leggett LK, Ahmed K, Vanier C, Sadik A (2021). A suggested strategy to integrate an elective on clinical nutrition with culinary medicine. Med Sci Educ.

[52] Magallanes E, Sen A, Siler M, Albin J (2021). Nutrition from the kitchen: culinary medicine impacts students’ counseling confidence. BMC Med Educ.

[53] Musick DW, Trinkle DB, Tabor J. Using a culinary health curriculum to teach teamwork skills: A new interprofessional education experience for medical, nursing and physician assistant students. J Res Interprof Pract Educ. 2020;21:100391.

[54] Jaroudi SS, Sessions WS, Wang VS, Shriver JL, Helekar AS, Santucci M, Cole L, Ruiz J, Fackrell J, Chauncey K (2018). Impact of culinary medicine elective on medical students’ culinary knowledge and skills. Proc (Bayl Univ Med Cent).

[55] Pang B, Memel Z, Diamant C, Clarke E, Chou S, Gregory H (2019). Culinary medicine and community partnership: hands-on culinary skills training to empower medical students to provide patient-centered nutrition education. Med Educ Online.

[56] Ring M, Cheung E, Mahadevan R, Folkens S, Edens N (2019). Cooking up health: a novel culinary medicine and service learning elective for health professional students. J Altern Complement Med (New York, NY).

[57] Rothman JM, Bilici N, Mergler B, Schumacher R, Mataraza-Desmond T, Booth M, Olshan M, Bailey M, Mascarenhas M, Duffy W (2020). a culinary medicine elective for clinically experienced medical students: a pilot study. J Altern Complement Med (New York, NY).

[58] D’Adamo CR, Workman K, Barnabic C, Retener N, Siaton B, Piedrahita G, et al. Culinary medicine training in core medical school curriculum improved medical student nutrition knowledge and confidence in providing nutrition counseling. Am J Lifestyle Med. 2021;155982762110217. 10.1177/15598276211021749 PMC9644147 36389046

[59] Vanderpool LE, Trilk JL, Griffin SF, Condrasky MD (2020). Culinary medicine an evaluation to assess the knowledge, attitudes, behaviors, and confidence of first-year medical students in a culinary medicine teaching kitchen. Top Clin Nutr.

[60] Vaccani J-P, Javidnia H, Humphrey-Murto S (2016). The effectiveness of webcast compared to live lectures as a teaching tool in medical school. Med Teach.

[61] Ibrahim ME, Al-Shahrani AM, Abdalla ME, Abubaker IM, Mohamed ME (2018). The effectiveness of problem-based learning in acquisition of knowledge, soft skills during basic and preclinical sciences: medical students’ points of view. Acta Inform Med.

[62] Bhogal SK, Murray MA, McLeod KM, Bergen A, Bath B, Menon A, Kho ME, Stacey D (2011). Using problem-based case studies to learn about knowledge translation interventions: an inside perspective. J Contin Educ Health Prof.

[63] Bennett S, Whitehead M, Eames S, Fleming J, Low S, Caldwell E (2016). Building capacity for knowledge translation in occupational therapy: learning through participatory action research. BMC Med Educ.

[64] Thandi CS, Forrest S, Williamson C (2016). The role of early inter-professional and inter-agency encounters in increasing students’ awareness of the clinical and community context of medicine. Perspect Med Educ.

[65] Mitchell LJ, Ball LE, Ross LJ, Barnes KA, Williams LT (2017). Effectiveness of dietetic consultations in primary health care: a systematic review of randomized controlled trials. J Acad Nutr Diet.

[66] Vos TP, Danaei GMD, Shibuya KP, Amann MP, Anderson HRP, Andrews KGMPH, Aryee MP, Bacchus LJP, Balmes JP, Barker-Collo SP (2013). A comparative risk assessment of burden of disease and injury attributable to 67 risk factors and risk factor clusters in 21 regions, 1990–2010: a systematic analysis for the Global Burden of Disease Study 2010. The Lancet.

[67] Evans D (2003). Hierarchy of evidence: a framework for ranking evidence evaluating healthcare interventions. J Clin Nurs.

