# Supplementary material for: Exploring culinary medicine as a promising method of nutritional education in medical school: a scoping review
Source: BMC Med Educ. 2022 Jun 7;22:441. doi: 10.1186/s12909-022-03449-w (PMC9175378; doi:10.1186/s12909-022-03449-w)
Supplement: Supplementary file 1 — Additional file 1. [file 12909_2022_3449_MOESM1_ESM.docx]

**Supplementary material:**

Searches entered for individual databases listed below:

**SAGE journals**

("culinary medicine" OR "nutritional medicine" OR "culinary education" OR "food as medicine") AND ("medical") AND (“student” OR "training" OR "education" OR "program")

**Taylor and Francis online:**

("Culinary Medicine" OR "Nutritional Medicine" OR "Culinary Education" OR "Food as medicine") AND (medical) AND (Student OR program OR education OR training)

**PROQUEST (Central) – “peer reviewed” selected**

("Culinary Medicine" OR "Nutritional Medicine" OR "Culinary Education" OR "Food as medicine") AND (medical) AND (Student OR program OR education OR training)

**MEDLINE (EBSCOhost) – “Scholarly (peer reviewed) journals” selected**

("Culinary Medicine" OR "Nutritional Medicine" OR "Culinary Education" OR "Food as medicine") AND (medical) AND (Student OR program OR education OR training)

**Web of science:**

("Culinary Medicine" OR "Nutritional Medicine" OR "Culinary Education" OR "Food as medicine") AND (medical) AND (Student OR program OR education OR training)

**SCOPUS:**

("Culinary Medicine" OR "Nutritional Medicine" OR "Culinary Education" OR "Food as medicine") AND (medical) AND (Student OR program OR education OR training)
