# Supplementary material for: Exploring culinary medicine as a promising method of nutritional education in medical school: a scoping review
Source: BMC Med Educ. 2022 Jun 7;22:441. doi: 10.1186/s12909-022-03449-w (PMC9175378; doi:10.1186/s12909-022-03449-w)
Supplement: Supplementary file 2 — Additional file 2. [file 12909_2022_3449_MOESM2_ESM.docx]

| **Table A1 – Example of Data Extraction “Table Charting”** | | | | | | |
| --- | --- | --- | --- | --- | --- | --- |
|  | **Lieffers 2021**- Interprofessional culinary education workshops at the University of Saskatchewan | **Magallanes 2021** - Nutrition from the kitchen: culinary medicine impacts students' counseling confidence | **Asano 2021** - A culinary medicine elective course incorporating lifestyle medicine for medical students | **Leggett 2021** - A suggested strategy to integrate an elective on clinical nutrition with culinary medicine | **Poulton 2021** - A taste of virtual culinary medicine and lifestyle medicine - an online course for medical students | **Hashimi 2020** - Cooking demonstrations to teach nutrition counseling and social determinants of health |
| TRENDS REPORTED IN RESULTS |  |  |  |  |  |  |
| (target population) |  |  |  |  |  |  |
| Did the study record population demographics? | no | yes | yes | yes | no | no |
| is the course an elective? | yes | yes | yes | yes | yes | yes |
| Was a participant recuitment method reported? | yes | yes | yes | yes | no | no |
| Method of recruitment? |  |  |  |  |  |  |
| Did the study report a sample size? | yes | yes | yes | yes | yes | yes |
| Did the study report attrition rates? | no | yes | yes | yes | yes | yes |
| Were the characteristics of those subjects choosing to participate and those who dropped out described? | no | yes | no | no | no | no |
| Did the intervention contrast practical nutrition education against traditional nutrition education? | no | no | yes | yes | no | no |
| Did the intervention focus on improving interprofessional and doctor patient relationships? | no | yes | no | no | yes | no |
| Did the intervention focus on nutritional counselling? | no | yes | yes | yes | yes | no |
| Did the intervention focus on nutritional attitudes? | no | yes | yes | yes | yes | no |
| Did the intervention focus on nutrition skills? | yes | no | yes | yes | yes | no |
| Did the intervention focus on nutritional knowledge? | yes | yes | yes | no | yes | no |
| Did the intervention focus on combatting chronic disease through nutrition? | no | no | yes | yes | no | no |
| Did the intervention focus on practical nutrition education in the medical curriculum? | yes | yes | yes | yes | no | yes |
| Did the study ask participants specifically, "do you think culinary medicine should be integrated in the medical curriculum" | no | no | no | no | no | no |
| Did the intervention display any positive outcomes with statistical signifiance? | no | yes | yes | yes | no | no |
| Did the intervention display any negative/nill outcomes? | no | yes | no | yes | no | no |
| did the study include a baseline survey (pre-intervention survey) | no | yes | yes | no | yes | no |
| Were any within-groups analyses conducted that allowed researchers to draw conclusions about how different subpopulations responded to an arm of the design? | no | no | no | no | no | no |
| Was the intervention delivered by a chef? | yes | no | yes | yes | no | no |
| Was the intervention delivered by a physcian? | no | yes | yes | no | yes | no |
| Was the intervention delivered by medical school faculty? | yes | no | yes | no | yes | yes |
| Was the intervention delivered by hospital dietitians? | no | yes | no | no | no | no |
| Was the intervention delivered by other medical students? | yes | no | no | no | no | no |
| Was the intervention delivered by nutrition faculty? | yes | no | no | yes | no | yes |
| Was the intervention adopted by multiple agencies/universities? | no | yes | no | no | no | no |
| Was the intervention performed in a teaching kitchen? | yes | yes | yes | no | no | no |
| Was the intervention performed in a university classroom / lecture halls? | no | no | yes | no | no | no |
| Was the intervention performed in community settings? | no | no | no | no | no | yes |
| Was the intervention performed in a regular / offsite kitchen? | no | no | no | yes | yes | no |
| Was the intervention delivered by patients/community members? | no | no | no | no | no | no |
| Was the intervention used an original curriculum? | yes | no | no | yes | no | yes |
| Was the intervention used an established curriculum? (CHOP) | no | yes - "health meets food" | no | no | no | no |
| Was the intervention used a modification of an established curriculum | no | no | yes- Health meets Food | no | yes | no |
| Did the intervention include case based discussions/ PBL? | yes | no | no | no | no | no |
| Did the intervention include collaborative cooking sessions? | yes | yes | yes | yes | yes | no |
| Did the intervention include pre course preperations (pre readings, videos, assignments) | no | no | yes | no | yes | no |
| Did the intervention include pre session quizs? | yes | no | yes | no | no | no |
| Did the intervention include hands on culinary skill lessons? | yes | yes | yes | yes | yes | yes |
| Did the intervention include after class assignments/ homework? | no | no | yes | no | no | no |
| Did the intervention include didactic sessions? | yes | no | yes | yes | no | no |
| Was the complete course equal to or greater than 25 contact hours? | no | no | yes | NR | no | no |
| Did the study report total costs of running the program | no | no | no | no | no | no |
| Did the study survey students' change in nutritional attitudes pre and post course | no | yes | yes | no | yes | no |
| if yes then, were they signficant? | - | yes | yes | - | no | - |
| Did the study survey students' change in culinary skill pre and post course | no | yes | no | no | no | no |
| if yes then, were they signficant? | - | yes | - | - | - | - |
| Did the study survey students' change in competency or confidence providing nutritional counselling pre and post post course | no | yes | yes | no | yes | no |
| if yes then, were they signficant? | - | yes | yes | - | no | - |
| Did the study survey students' change in personal health behaviours pre and post course | no | yes | no | no | yes | no |
| if yes then, were they signficant? | - | yes | - | - | no | - |
| Did the study survey changes in students' ability to identify food by visual inspection pre and post course | no | no | no | no | no | no |
| if yes then, were they signficant? | - | - | - | - | - | - |
| Did the study collect student evaluation of the course after completion | yes | no | yes | yes | yes | yes |
| if yes then, were they signficant? | - | - | yes | yes | no | - |
| Did the study report observations from course facilitators after the completion of the course | no | no | no | no | no | no |
| if yes then, were they signficant? | - | - | - | - | - | - |
| Did the study report statistical significance of changes seen pre- and post course | no | yes | yes | no | no | no |
| if yes then, were they signficant? | - | yes | yes | - | - | - |
| Did the study use thematic analysis to evaluate findings | no | no | no | no | no | yes |
| if yes then, were they signficant? | - |  | - | - | - | no |
| Did the study use odds ratios to evaluate findings | no | yes | no | no | no | no |
| if yes then, were they signficant? | - | yes | - | - | - | - |
| Did the study use a self generated competency scale | no | no | no | no | no | no |
| Did the study use a verified competency scale | - | yes | yes | yes | no | yes |
| Was there any cost-effectiveness or cost-benefit analysis performed/reported? | no | no | no | no | no | no |
| Was any changes or modifications of the intervention in any way during the course of the study reported? | no | no | no | no | no | yes |
| did the study follow up with participants after completion of the course? (aka did they really improve in nutritional couselling as physicians?) | no | no | no | no | no | no |
| Is the intervention still in place? | yes | no | yes | no | no | no |
